# A deep continental aquifer downhole sampler for microbiological studies

**DOI:** 10.3389/fmicb.2022.1012400

**Published:** 2023-01-04

**Authors:** Magali Ranchou-Peyruse, Marion Guignard, Perla G. Haddad, Sylvain Robin, Fabrice Boesch, Maud Lanot, Hervé Carrier, David Dequidt, Pierre Chiquet, Guilhem Caumette, Pierre Cézac, Anthony Ranchou-Peyruse

**Affiliations:** ^1^E2S-UPPA, CNRS, IPREM, Universite de Pau & Pays Adour, Pau, France; ^2^E2S-UPPA, LaTEP, Universite de Pau & Pays Adour, Pau, France; ^3^Joint Laboratory SEnGA, E2S-UPPA-Teréga, Pau, France; ^4^Modis, Pau, France; ^5^E2S-UPPA, CNRS, TOTAL, LFCR, Universite de Pau & Pays Adour, Pau, France; ^6^STORENGY – Geosciences Department, Bois-Colombes, France; ^7^Teréga, Pau, France

**Keywords:** deep aquifer, microorganisms, downhole sampler, UGS, ATEX, geological storage

## Abstract

To be effective, microbiological studies of deep aquifers must be free from surface microbial contaminants and from infrastructures allowing access to formation water (wellheads, well completions). Many microbiological studies are based on water samples obtained after rinsing a well without guaranteeing the absence of contaminants from the biofilm development in the pipes. The protocol described in this paper presents the adaptation, preparation, sterilization and deployment of a commercial downhole sampler (PDSshort, Leutert, Germany) for the microbiological studying of deep aquifers. The ATEX sampler (i.e., explosive atmospheres) can be deployed for geological gas storage (methane, hydrogen). To validate our procedure and confirm the need to use such a device, cell counting and bacterial taxonomic diversity based on high-throughput sequencing for different water samples taken at the wellhead or at depth using the downhole sampler were compared and discussed. The results show that even after extensive rinsing (7 bore volumes), the water collected at the wellhead was not free of microbial contaminants, as shown by beta-diversity analysis. The downhole sampler procedure was the only way to ensure the purity of the formation water samples from the microbiological point of view. In addition, the downhole sampler allowed the formation water and the autochthonous microbial community to be maintained at *in situ* pressure for laboratory analysis. The prevention of the contamination of the sample and the preservation of its representativeness are key to guaranteeing the best interpretations and understanding of the functioning of the deep biosphere.

## Introduction

The latest estimates of the volume represented by all the world's aquifers included in the first two kilometers of the crust reach 22.6 million km^3^, while the total volume of fresh surface water is estimated to be only 100,000 km^3^ (Gleeson et al., 2015). In 2018, Magnabosco and her collaborators published a review compiling microbial concentration and diversity data from 3,800 continental subsurface studies, a third of which were groundwater studies. The authors estimated that this deep continental biomass could represent 23 to 31 petagrams of carbon (C), hundreds of times more than that comprised by the total of humanity. Approximately 98% of the world’s freshwater reserves are located in aquifers (Margat and van der Gun, 2013). Shallow, deep, freshwater or saline aquifer resources must be managed, as they play strategic roles in our societies in terms of both water resources (drinking water, irrigation) and participating in energy transitions (energy storage through underground gas storage (UGS) and geothermal energy) and carbon sequestration in greenhouse gas reduction approaches (De Silva and Ranjith, 2012; Sainz-Garcia et al., 2017; Limberger et al., 2018).

The microorganisms present in aquifers, whether indigenous or nonindigenous, can be involved in natural or stimulated bioattenuation processes (with hydrocarbons, pesticides, chlorinated solvents, etc.) and can be used to reduce the concentrations of contaminants, such as nitrate (Calderer et al., 2010; Matteucci et al., 2015; Lueders, 2017; Aldas-Vargas et al., 2021). The Deep Carbon Observatory program^1^ has clearly shown that a detailed and exhaustive understanding of the carbon cycle necessitates these environments being taken into account. It is obvious that studies on the impacts of climate change on these ecosystems, including deep aquifers, will proliferate in the coming years. The topics of interest are diverse and may include studies on decreases in water reserves and their quality, bioattenuation and facilitated biodegradation, and the impacts of artificial groundwater recharge activities (Russo and Lall, 2017; Abdelmohsen et al., 2019; Jasrotia et al., 2019; Ranchou-Peyruse et al., 2021). Sampling is the starting point for all microbiological studies and therefore is a key step that is a difficult challenge in deep environments (Lehman, 2007; Mangelsdorf and Kallmeyer, 2010; Wilkins et al., 2014; Cario et al., 2019; Soares et al., 2019).

Unlike surface ecosystems, the study of continental aquifers in general, especially those several hundreds of meters deep, is complicated because of access-related difficulties, safety and sparse sampling sites, which often involve water sampling *via* drilled wells. These studies involve the withdrawal of formation water *via* surface installations, wellheads, or control or operating wells. For the deepest aquifers, these steel pipes can exceed 1 km in depth and represent important sites that can be characterized as “windows” into deep environments (Sorensen et al., 2013; Kadnikov et al., 2018). Conditions in these pipes are different from those in aquifers: these are open ecosystems rather than microporous ecosystems, with the possible presence of oxygen, metal alloys, pipe maintenance grease, and wellhead contamination by surface ecosystems (Basso et al., 2005; Lehman, 2007; Korbel et al., 2017; Hershey et al., 2018; Mullin et al., 2020). To limit the microbial contamination inherent in a well, most standard sampling protocols involve at least a purge of 1 (Garvis and Stuermer, 1980; Stevens et al., 1993), 2 (Pionke and Urban, 1987), or even 3 to 5 bore volumes (Sundaram et al., 2009; Hose and Lategan, 2012; Smith et al., 2012, 2015; Gründger et al., 2015; Hershey et al., 2018) to remove stagnant water. The use of a pump is necessary when the well is not eruptive (López-Archilla et al., 2007; Kirs et al., 2020). Some researchers have introduced a polyamide tube factory cleaned with a back-pressure valve at the lower end of the tubes to prevent water from flowing out during recovery (Nurmi and Kukkonen, 1986; Itävaara et al., 2011). The tube was made up of several sections sized 100 meters each to avoid contamination from the well. This method of sampling could be associated with inflatable packers to isolate specific fracture zones (Purkamo et al., 2013) but cannot be used to collect water from a monitoring well in the context of a gas storage aquifer for safety reasons. In 2005, Basso and her coauthors published a study on the procedure for cleaning an 800-meter-deep well before sampling the formation water to study microbial diversity. This work has shown that purging alone, regardless of its duration, cannot guarantee that the samples are uncontaminated by the microorganisms developing in biofilms colonizing the steel surfaces inside the well, valve seals and grease used for wellhead maintenance. The proposed procedure first involved a very large purge throughout the various stages with more than 25 times the volume of the tubing. Mechanical cleaning was carried out to remove the biofilms. Finally, three volumes of chlorine (4 liters of 9.6% active chlorine solution each) were injected into the bottom of the well before being eliminated by purge water, thus preventing injection into the aquifer. This procedure was applied at 11 different sites and gave rise to several published research works (Basso et al., 2005, 2009; Klouche et al., 2009; Berlendis et al., 2010; Aüllo et al., 2016; Ranchou-Peyruse et al., 2017; Godin et al., 2020; Ranchou-Peyruse et al., 2021). In addition to the quality of the samples, this last technique has the advantage of having no restrictions on the volume of water to be withdrawn after the well cleaning protocol is completed. Although this procedure is very effective since it drastically limits the risk of contamination, it is very complex and time-consuming and must be established at the site (Wireline Combi Unit, evacuation of purged water, etc.). As a result, it requires very close collaboration with the field operator and a substantial but necessary financial investment. It is therefore necessary to develop a sampling method that is just as effective but less restrictive. Downhole-sampling approaches appear to be the most relevant for ensuring the noncontamination of water samples and their associated microbial communities. The use of a downhole sampler is common in the field of geosciences in the broad sense for collecting samples of fluids such as water, dissolved or undissolved gases and oils to analyze their physicochemical or isotopic compositions (Rivard et al., 2018; Struchkov and Rogachev, 2018; Banks et al., 2019; Osselin et al., 2019). In a UGS context, the study of microbial communities from geological reservoirs represents a real challenge because of the hundreds of meters of pipe, pressure, potential toxicities of gases (methane, sulfide, carbon dioxide, etc.), and explosive atmosphere of the study environment.

Here, a new sampling procedure with a commercial downhole sampler (Positive Displacement Sampler, PDSshort, Leutert, Germany) is presented. This sampler was previously used to sample deep wells as far as 4,240 mbs (meters below the surface) to maintain fluid samples at an *in situ* pressure to study the isotopic compositions of saline formation waters and dissolved gases (Regenspurg et al., 2010; Kietäväinen et al., 2012; Kampman et al., 2013; Feldbusch et al., 2018). There are a few examples of sampling deep continental aquifers with downhole samplers, but the procedures are not detailed and are difficult to replicate. An older sampler model (Sampler Model 60', Leutert) was employed to study the microbial community evolving in the formation water of an aquifer used for the storage of town gas in Lobodice (Czech Republic) in the early 1990s (Šmigáň et al., 1990). From the end of the 1990s, a similar pressurized groundwater sampling instrument called “PAVE” developed by Anttila et al. (1999) was used to study the microbiology of deep igneous rock aquifers in Finland (Haveman et al., 1999; Haveman and Pedersen, 2002; Öhberg, 2006; Pedersen et al., 2008). This wire-line PAVE downhole equipment used in deep biosphere studies in Fennoscandian bedrock had a small sampling volume (150 to 250 ml, Haveman et al., 1999; Haveman and Pedersen, 2002; Öhberg, 2006; Pedersen et al., 2008). However, deep aquifers are most often oligotrophic environments with microbial concentrations that are often very low and on the order of 10^1^ to 10^5^ cells.ml^–1^ (Lerm et al., 2013; Bomberg et al., 2015, 2016; Magnabosco et al., 2018). These low concentrations necessitate an ability to work with the highest possible volumes. In addition, experiments in a pressurized reactor aimed at simulating these environments over periods of several months require volumes of more than 1 L of water, i.e., two samplers in the context of the study of Haddad and collaborators (Haddad et al., 2022a, b). Moreover, the electric current presents a danger during PAVE system deployment in an explosive atmosphere and prohibits its use on aquifers used to store natural gas (Ruotsalainen and Snellman, 1996). Later, to study the impact of CO_2_ injection on microbial communities, a downhole sampler (Doppelkugelbüchse, DKB) was deployed in a saline aquifer at 675 mbs (Morozova et al., 2013).

In this article, we present the procedure for preparing and deploying a downhole sampler to sample water from a deep aquifer while respecting microbiological conditions such as anoxia, sterility and pressure maintenance. We recently used this sampler in two sampling campaigns related to geological gas storage and their interactions with autochthonous microbial communities (Haddad et al., 2022a, b). Here, the samples were taken from a monitoring well of a deep aquifer (–582 mbs; 69 bar; 36°C) used for the storage of natural gas. The formation water was maintained at the *in situ* pressure from the aquifer to the laboratory before undergoing controlled depressurization. To validate our procedure and prove its importance, basic but fundamental microbiological analyses were carried out: (i) the physiological state of the microbial cells was assessed by epifluorescence microscopy approaches and (ii) the bacterial taxonomic diversity was monitored at the wellhead throughout the sampling operation, as well as in the water collected using the two downhole samplers sent successively to the bottom of the well.

## Materials and methods

### Sampling site

Water was sampled from the AB_L_1 control well of an aquifer used for the storage of natural gas in the Aquitaine geological basin (southwest France) in January 2019. This well has already been used to study microbial diversity in formation water (Ranchou-Peyruse et al., 2019; Haddad et al., 2022a). The tubing of the monitoring well descends to a depth of 582 mbs and has an estimated volume of 7.6 m^3^. No water from the upper formation can penetrate inside the tubing. The formation water evolves at a geological level dating from the Eocene-Lutetian that consists of inframolassic sands with sandy detrital facies (sandstone to numulites) at 36°C. The pressure at the bottom was estimated to be approximately 69 bar and fluctuated depending on the storage gas in place, reaching 3 GNm^3^. At the time of sampling, the well was eruptive, and the stored gas was located approximately 250 meters from the well in the inframollassic sands. Although the sampler can be deployed in nonartesian drillholes, a purge water flow rate of 10 m^3^.h^–1^ was maintained to guarantee that the samples would not be contaminated by pieces of biofilm torn from the surface of the pipe during the descent of the sampler. During sampling, two downhole samplers were deployed consecutively to obtain formation water with representative autochthonous microbial communities. Simultaneously, several water samples were collected at the wellhead.

### Description of the downhole sampler

**Figure 1:**
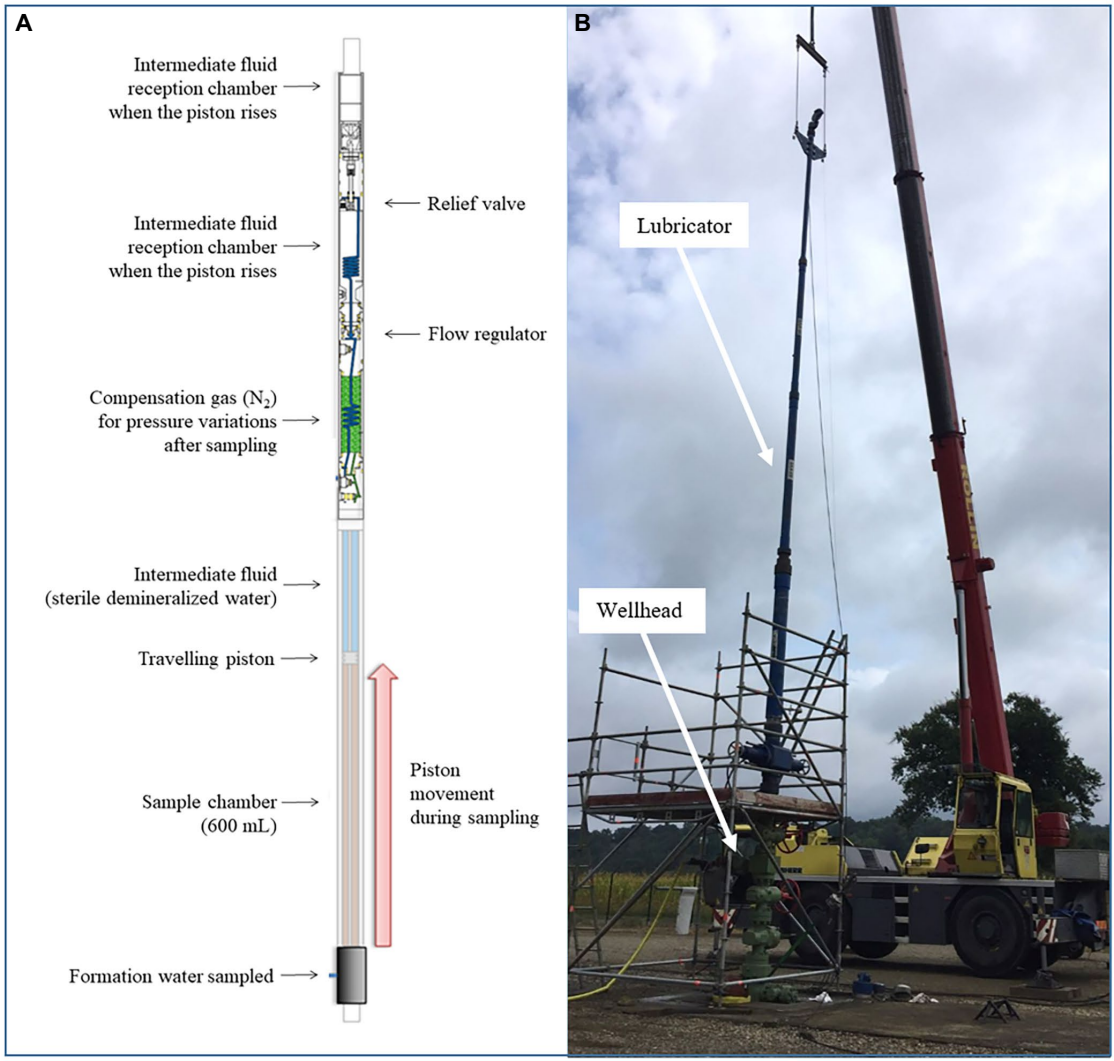
Use of the LEUTERT OPS downhole sampler to collect microorganisms from a deep aquifer. **(A)** Descriptive diagram of the downhole sampler; **(B)** Deployment of the sampler on site: the sampler is inserted into a chamber. Then, the chamber is connected to the wellhead before it is opened, and the sampler descends into the well.

A LEUTERT One Phase Sampler OPS sampler (Adendorf, Germany) was used during a one-day sampling campaign (Figure 1A) to avoid contamination by biofilms developing on the surfaces of the well, preserve anoxic conditions and maintain the *in situ* pressure during the process. With a length of 4.63 m and a diameter of 43 mm, the sampler is made of stainless steel according to NACE MR-01-75 and a bronze alloy. It operates up to 1,035 bar and 180°C (supplier information). Fortunately, this sampler has a steam-sterilizable sampling chamber that opens and closes only at the depth to be sampled, thus making it possible to obtain a representative sample of the target microbial environment. The sampler is ATEX-certified and can be used in explosive atmospheres (ATEX). Indeed, it is completely mechanical and lacks any source that can generate a spark or energy that can cause an explosion. Practically, the sampler is composed of three compartments: (i) The upper compartment is filled with a biologically inert gas such as nitrogen (Linde) during this sampling, and it is mechanically isolated from the other compartments of the tool until the piston, at the end of its stroke (i.e., at the end of sampling), activates a set of linkages allowing the following actions: closure of the sample chamber, sealing of the connection between the intermediate fluid reception chamber and the sample compartment, and connection of the nitrogen compartment with the sample compartment *via* the traveling piston. This latter connection allows the fluid in the nitrogen compartment to exert a pressure force on the downstream face of the piston once the sample has been taken. This force ensures that the sample is maintained at a minimum pressure close to the pressure imposed on the nitrogen compartment at the surface (i.e., 69 bar). (ii) The intermediate fluid keeps the piston in its low position until the opening of the channel connecting the sample compartment and the sterile demineralized water compartment. This connection is made mechanically at the end of the countdown programmed on the clock/mechanical actuator. (iii) The lower compartment (i.e., sampling chamber), 600 cm^3^, was used to collect the water sample. A flow regulator adds a calibrated pressure drop to control the transfer rate and therefore the speed of sample collection. When the sampled fluid occupies the entire dedicated compartment, i.e., 600 cm^3^, the compartment containing nitrogen and the intermediate fluid are mechanically isolated from the other components of the tool. During ascent and once at the surface, the bottom pressure is maintained inside the sampler with a slight modification linked to the change in temperature.

### Preparation of the sampler for microbiological studies

In the laboratory, all of the instruments that would come in contact with the collected sample were cleaned, degreased and disinfected with 70% ethanol. Each operation that could lead to contamination from the external environment was subject to cleaning with alcohol and precautions to avoid microbial contamination until the equipment was deployed in the well. This was followed by a step of rinsing all the parts with sterile water. The water collection chamber, filled with 50 ml of distilled water, was then sterilized for 2 h at 125°C using an enveloping jacket with heating resistance (Supplementary Figure S1). Finally, the timer released a mechanical system allowing the piston to go up. The latter triggered the opening of the pressurized gas responsible for maintaining the pressure of the collected sample and at the same time the closing of the inlet orifices of the sampling chamber. The operation settings allowed us to work with time steps from 15 min to 5 h, but the equipment could be reprogrammed to go up to 24 h.

### Field deployment

The downhole sampler was operated by Modis (Pau, France). To allow the descent of the sampler with a crane along the monitoring well, the downhole sampler was first inserted into a lubricator (Figure 1B), and centralizers were positioned to prevent the sampler from coming into contact with the internal surface of the lubricator and the well at all times during descent. Once the sampler was lowered to the desired depth, the fluid to be sampled exerted a pressure force on the upstream surface of the piston. During this sampling campaign, two downhole samplers were used approximately 2 h apart. Back in the laboratory, the water samples could be slowly depressurized to be analyzed, as was done in this experiment.

### Depressurization of the sample

All connections, tubes and flasks that were in contact with the water collection chamber and with the collected water were sterilized beforehand *via* steam sterilization, high temperature overnight or cleaning with 70% ethanol. Because deep aquifers are anoxic environments, the air inside the sterilized flasks used to collect water containing microorganisms was replaced by nitrogen. To obtain as representative of a sample as possible, the rate of depressurization had to be controlled to avoid lysing the microbial cells. To this end, a pressure gauge was connected to the filling opening of the nitrogen chamber, and a pressure compensation system consisting of a sterilized high-pressure manual pump equipped with a pressure gauge and one two-way valve was used (Supplementary Figure S2). This setup ensured an equal pressure when the sampler was connected to the transfer tubing, and the pressure drop was controlled while the sample was depressurized. The valve of the outlet of the sampling chamber was first slowly opened to balance the two upstream/downstream pressure taps without creating a depressurization shock that could lead to the death of the microorganisms in the sample. The gradual purging of nitrogen was carried out from the transfer cylinder pressure compensation chamber. Once the sample pressure was reduced to approximately 5 bar, it was very gradually brought to atmospheric pressure during its transfer into sterile glass bottles equipped with overpressure exhaust filters.

### Biomass filtration and nucleic acid extraction

Some of the samples collected with the both downhole samplers during this campaign (2 × 500 ml) were kept separately in order to simulate the conditions of the deep aquifer inside a high-pressure reactor (Haddad et al., 2022a). Here, 100 ml of the formation waters sampled with each of the two downhole samplers (DS1 and DS2) and four 1 L flasks of the wellhead waters (WHS1 to WHS4) were filtered with 0.22 μm porosity filters (cellulose nitrate filter, Sartorius Stedim) for microbial community taxonomic diversity analyses. Subsequently, the eluate was filtered a second time with 0.1 μm porosity filters (polyethersulfone filter, Sartorius Stedim) to collect cells smaller than 0.22 μm. Each filter was stored at-80°C for future use to conserve nucleic acids. The filters were then ground in a mortar with liquid nitrogen. All of the nucleic acids were extracted with the Fast RNA Pro-Soil (MP Bio) kit following the manufacturer's instructions until the nucleic acids were eluted in 50 μl of DEPC water included in the kit. Then, the extracted DNA and RNA were separated with the All Prep DNA/RNA kit (QIAGEN). For this study, we were only interested in DNA analysis. The DNA concentrations were quantified with a Qubit fluorometer (Invitrogen by Life Technologies, Carlsbad, CA). DNA concentrations were not detectable for the 100 ml samples from the downhole samplers.

### Nested PCR and high-throughput sequencing

A conventional PCR approach did not allow sufficient amplification of all samples. For each sample, the V3-V4 region of the 16S rRNA gene was amplified by nested PCR (Yu et al., 2015) with the PCR CORE kit (Roche). The primers used to target this region were 8F/1489R (Weisburg et al., 1991), 344F_5’–ACGGRAGGCAGCAG-3’ and 801R_5’-CGGCGTGGACTTCCAGGGTATC-3’ (Simmon et al., 2006; Wichels et al., 2006). The first amplification was carried out as follows: a strand separation at 94°C for 2 min; 15 cycles of 94°C for 40 s; annealing at 55°C for 40 s; and strand extension at 72°C for 45 s. A final stage of 7 min at 72°C for further strand extension. The second amplification differed in the 30 amplification cycles: 94°C for 30 s; 65°C for 30 s; 72°C for 40 s. The 344F/801R primers contained the adapters 5’-CTTTCCCTACACGACGCTCTTCCGATCT-3’ and 3’-GGAGTTCAGACGTGTGCTCTTCCGATCT-5’ to achieve high-throughput sequencing. High-throughput sequencing was performed with a GenoToul genomic platform (Toulouse, France) that used MiSeq Illumina 2 × 250 bp technology, according to the manufacturer's protocol. The raw sequencing data were deposited in the NCBI SRA under bioproject ID PRJNA769063. The sequencing data were then checked for their quality and processed *via* the FROGS analysis pipeline developed by the GenoToul genomic platform in the Galaxy interface (Escudié et al., 2018) using Flash to merge the paired-end reads (Magoc and Salzberg, 2011), Swarm for sequence clustering based on the Sellers’ evolutionary distance (Sellers, 1974; Mahé et al., 2014), and VSEARCH with the *de novo* UCHIME method to eliminate chimeras (Edgar et al., 2011; Rognes et al., 2016). From the initial 518,289 reads, the pre-processing and filtration steps led to 409,781 reads. Rarefaction curves obtained for each sample (data not shown) indicated that sequencing was deep enough to estimate microbial composition and there did not seem to be any effect of filtration of different volumes of water between WHS and DS. After normalization, there were 20,682 reads per sample. The processed dataset was analyzed using the R “phyloseq” package (McMurdie and Holmes, 2013). The graphs were constructed using the R “ggplot2” package (Wickham, 2009). Alpha-diversity indices were calculated and comparison of microbial compositions between samples was obtained by performing a hierarchical clustering using the Ward D2 method and based on Jaccard’s analysis of beta-diversity. A heatmap representation was also generated. The taxonomic classification was based on the Silva database (version 138.1).

### Cell counts and microscopy

From all the water collected (samplers and well-head), the proportions of living and dead cells were determined by epifluorescence microscopy with the LIVE/DEAD BacLight Bacterial Viability Kit (Thermo Fisher Scientific), as described by the supplier. Briefly, 1 ml of water was spiked with 1.5 μl of SYTO9 and 1.5 μl of propidium iodide. After incubation in the dark for 15 min, the water was filtered through 0.2 μm pore-size black polycarbonate (Millipore) under vacuum as described in Ranchou-Peyruse et al. (2021). In each of the measurements, 20 randomly selected fields were observed, and 85 to 3,300 microbial cells were counted. To our knowledge, there are no black polycarbonate filters with a porosity of 0.1 μm, which explains the absence of this analysis. A Zeiss Observer.Z1c epifluorescence microscope equipped with a mercury light source was used.

## Results and discussion

### A tool adapted for deep aquifer sampling in the UGS context

Before being deployed on site, several laboratory tests were carried out to verify the maintenance of the sterility of the sampler and its proper functioning under pressures ranging from 50 to 100 bar. This pressure range was selected because it corresponds to French gas storage in deep aquifers (–500 mbs to –1,200 mbs). This tool was designed to be able to operate at 1035 bar in a petroleum environment. We successfully tested its operation at lower pressures without the use of grease (which is a source of contamination and exogenous molecules) for the moving parts in contact with the sampling chamber. Here, the downhole sampler was lowered in tubing with a minimum internal diameter of 69 mm, but it can reasonably be used down to 55 mm. The first tests were carried out without the use of centralizers and led to the scraping of the pipe during the descent and the contamination of the inlet to the sampling chamber (data not shown). We deduce that the use of such downhole samplers in inclined drillholes could compromise the quality of the microbiological sampling. In this type of well, the sampler, the slickline train and the cable continuously rub against the tubing.

### Control of the depressurization rate

**Figure 2:**
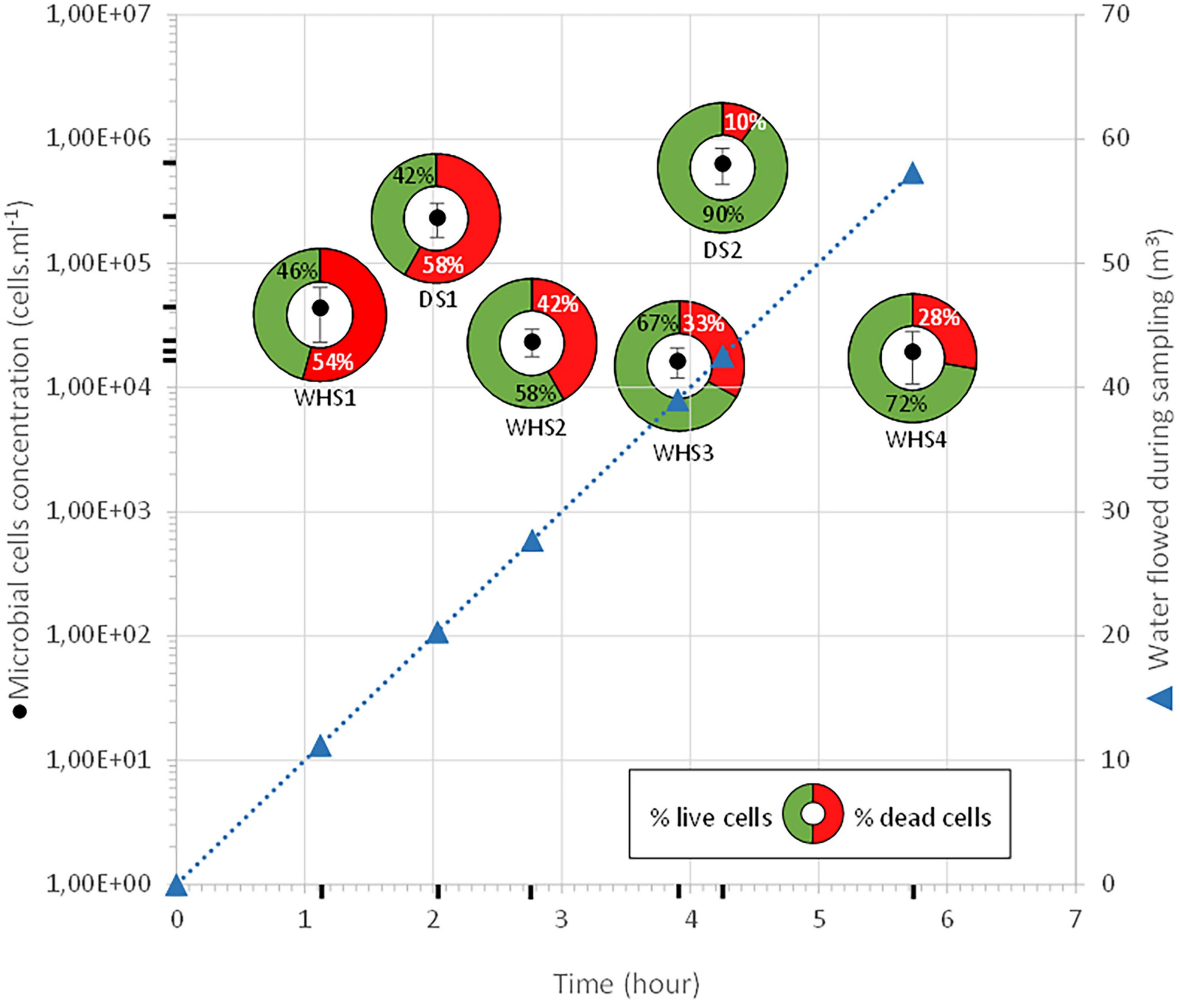
Enumeration and cell survival during the sampling campaign. The black circles represent total cell counts. For each of these counts, the cells with intact membranes, qualified as living cells, are represented in green, while the cells with lysed membranes, qualified as dead cells, are shown in red. As the well is artesian throughout the procedure, the volume of water purged is represented with blue triangles. DS: downhole sampling; WHS: wellhead sampling.

Throughout our operation, the monitoring well was in eruptive conditions with a water flow rate maintained at 10 m^3^.h^–1^, allowing the evacuation of any piece of biofilm or any particle possibly torn off during the descent of the sampler. Taking into account the flow rate as well as the volume of the well, raising a microbial cell out of the deep aquifer from 582 mbs represents a depressurization of 60 bar at atmospheric pressure in 46 min, or 1.3 bar.min^–1^. It is still difficult to assess the impact of this depressurization on the physiological state of microorganisms. This depressurization could explain the high percentages of dead cells, ranging from 28 to 54% of the community in samples WHS1, 2, 3 and 4 (WHS, wellhead sampling; Figure 2). However, in a study on MEOR (Microbial Enhanced Oil Recovery), the authors considered that with a depressurization of 1 bar.min^–1^, the deleterious effect on the cells was not “pronounced” (Krüger et al., 2016). Nevertheless, it should be noted the depressurization seems to have been made by stages of several hours, even days. Sampling with these downhole samplers enabled us to maintain the pressure and/or regulate the pressure decrease. In this work, the chosen depressurization speeds were lower than those experienced during wellhead sampling, with one relatively close (DS, downhole sampling; DS1: 1 bar.min^–1^) and the other significantly lower (DS2: ≈0.2 bar.min^–1^). The higher cell mortality in the case of the DS1 sampler supports this first hypothesis, and a comparison with the results obtained in the case of DS2 validates a depressurization rate of approximately 0.2 bar.min^–1^. Likewise, the lowest apparent cellular concentrations for water taken directly from the wellhead (between 1.6·10^4^ ± 4.4·10^3^ cell.mL^–1^ and 4.4·10^4^ ± 2.0·10^4^ cell.mL^–1^) compared to those taken with the sampler (DS1: 2.3·10^5^ ± 7.2·10^4^ cell.mL^–1^ and DS2: 6.3.10^5^ ± 2.0·10^5^ cell.mL^–1^) support the idea that the lysis of the cell membrane is more sensitive to depressurization, which could have led to the dispersion of genomic material outside the cell, making it impossible to count these cells by fluorescent nucleic acid stains (SYTO9 and propidium iodide).

### Samples free from microbial contamination

**Figure 3:**
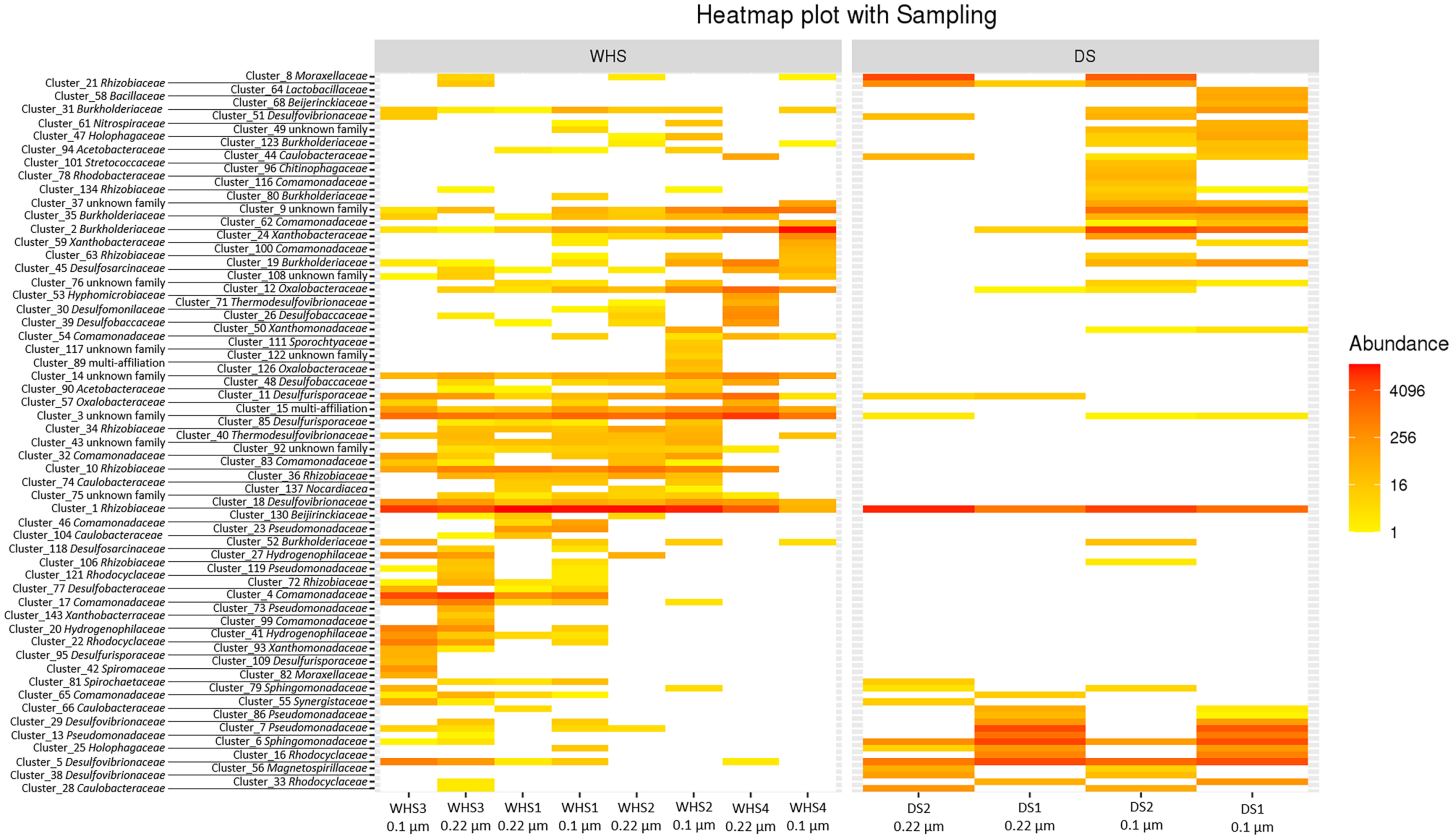
Heatmap showing the distributions of 108 bacterial OTUs in the formation water collected at the wellhead (WHS) and with the downhole samplers (DS) after a biomass concentration with filters with a porosity of 0.22 μm and 0.1 μm from the 0.22 μm-eluate. The taxa associated with the OTUs are presented to the left of the figure and in Supplementary Table S1.

**Figure 4:**
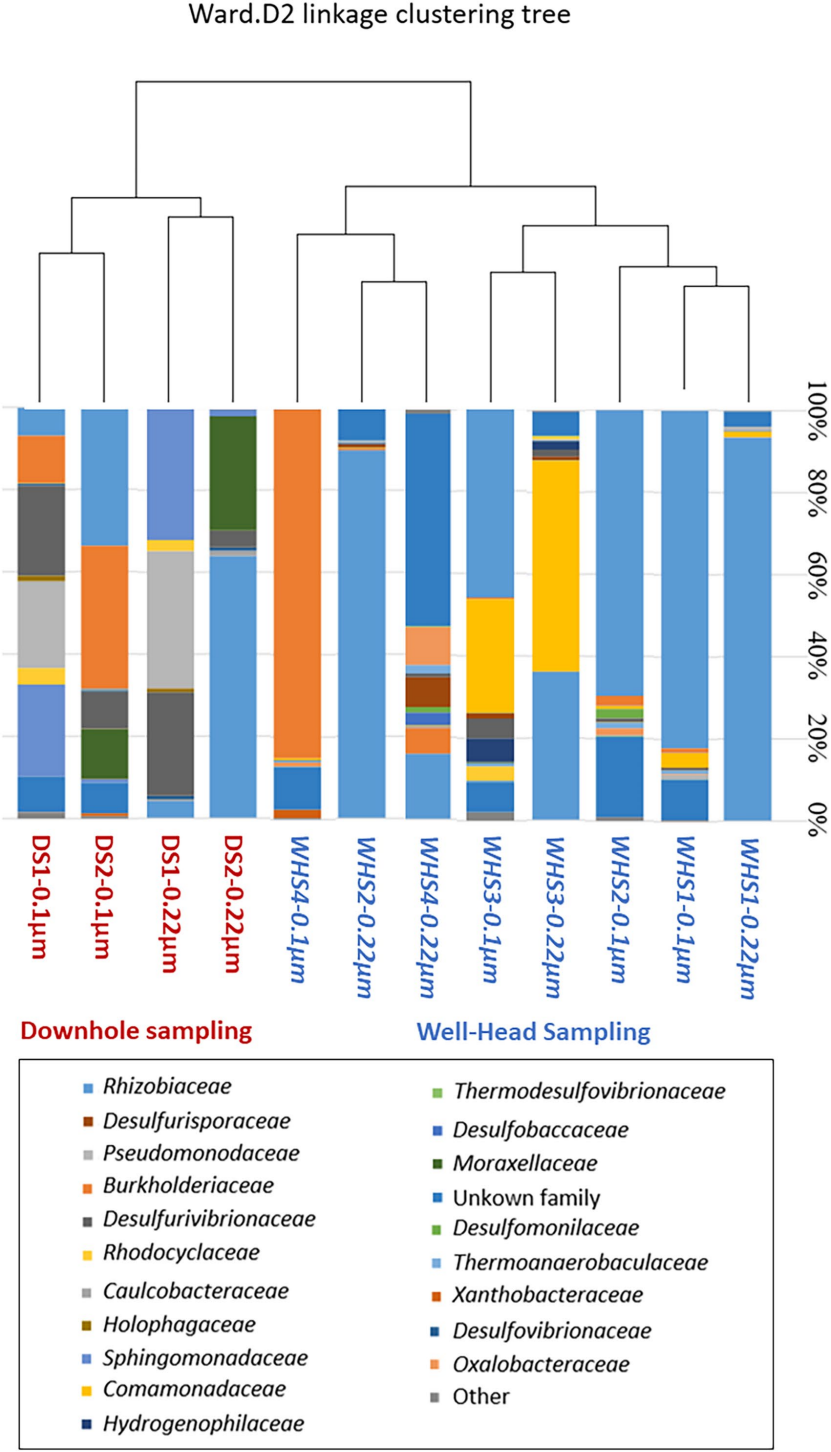
Jaccard’s analysis of the beta diversity and comparison representation of the dominant bacterial families based on the 16S rRNA gene (v3-v4) in different formation water samples from the downhole sampler (DS, red) and the wellhead sampler (WHS, blue). The deep aquifer bacterial community was obtained by 0.22 μm filtration or 0.1 μm filtration from the 0.22 μm eluate.

Regarding the water sampled at the wellhead, the water flow before sampling corresponded to more than 1 (1 × 7.6 m^3^ of well volume < 11.3 m^3^ of water discharged), 3 (22.8 m^3^ < 27.7 m^3^), 5 (38 m^3^ < 39 m^3^) and 7 (53.6 m^3^ < 57.3 m^3^) bore volumes for WHS1, WHS2, WHS3 and WHS4, respectively (Figure 2). The alpha-diversity of the microbial communities from the water sampled at the well-head decreased as the bore volumes increased, with an exception for WHS3 0.22 μm (Table 1). The differences in various metrics (number of observed OTU, Chao1 index, Shannon index, InvSimpson index) between the biomass filtered at 0.22 μm and 0.1 μm suggested the presence of a sub-community of smaller cell size (< 0.1 μm; 26 OTU in Figure 3). Despite the volumes of water discharged throughout the sampling day, the beta-diversity analysis clearly shows a difference between the microbial communities sampled at the wellhead and those sampled using the downhole sampler (Figure 4), even after 7 bore volumes were discharged, as for the WHS4 sample. These results clearly show that the simple water purges described in many studies of deep aquifers may not be sufficient to prevent contamination and therefore do not guarantee the purity of the microbial communities studied (Garvis and Stuermer, 1980; Pionke and Urban, 1987; Stevens et al., 1993; Sundaram et al., 2009; Hose and Lategan, 2012; Smith et al., 2012, 2015; Gründger et al., 2015; Hershey et al., 2018). In all cases, an evolution of the microbial taxonomic diversity was revealed during the withdrawal of the formation water (Table 1; Figure 4). We interpret this by the heterogeneity of the aquifer close to the well, discussed in more detail below. The low microbial concentrations often encountered in oligotrophic deep aquifer water make it necessary to filter the biomass to concentrate it for molecular or even culturing approaches (Basso et al., 2005; Trias et al., 2017; Guðmundsdóttir et al., 2019). Living in a deep environment promotes cell shrinkage to reduce the metabolic needs of microorganisms in an environmental context of extreme oligotrophy (Miyoshi et al., 2005; Luef et al., 2015; Wu et al., 2016). The use of filters with a porosity of 0.1 μm is recommended but is not necessarily easy when filtering water that may be clogged with mineral particles, in particular iron sulfides, which can be present in abundance in this kind of anoxic environment rich in ferrous iron and sulfide leading to precipitates such as pyrite. By continuing to rely on betadiversity, there was no difference found between the microbial communities according to the filtration porosity from the water collected at the wellhead (Figure 4). On the other hand, there seems to be an effect of the filter porosity in the case of the bacterial communities from the water sampled *via* the downhole samplers (DS), suggesting that the microbial contamination from the pipe surface (WHS) masks a possible cell size effect. Regarding the microbial communities, an analysis of the different taxonomic diversities based on the 16S rRNA gene by heatmap representation (Figure 3) and a comparison of the 20 dominant bacterial families (Figure 4) show variability between the different waters sampled at the wellhead (WHS) and in the samplers (DS). These differences could be explained by the expected heterogeneity of the microbial communities throughout the sampling (Goldscheider et al., 2006). The studied aquifer, which is homogeneous in its overall constitution and characterized by inframolassic sands, presents heterogeneity on a more local scale, with the presence of calcite, clay and iron sulfides (Haddad et al., 2022a). Obviously, the longer the sampling and the greater the volume discharged, the further the sampled bacterial populations are from the well. With a flow rate of 10 m^3^.h^–1^, the formation water circulating in the sands of the aquifer, with a porosity between 25 and 35% (Ranchou-Peyruse et al., 2019), can come from several meters around the well, approximately 3 to 4 m, which can be represented by imagining a sphere of influence centered on the strainers at the bottom of the well. With 108 operational taxonomic units (OTUs) present in at least one analyzed sample, it appears that there are 54 OTUs that are only found in the water sampled at the wellhead and not in the samplers (Figures 3, 4). These OTUs were mainly distributed among 6 bacterial families: *Desulfobaccaceae*, *Desulfomonilaceae*, *Hydrogenophilaceae*, *Thermoanaerobaculaceae*, *Thermodesulfovibrionaceae*, and *Xanthobacteraceae*. Three of these families are sulfate reducers (*Desulfobaccaceae*, *Desulfomonilaceae,* and *Thermodesulfovibrionaceae*), and one family includes bacteria exhibiting fermentation metabolism (*Thermonaerobaculaceae*). Mesothermal conditions raise questions about the presence of bacteria classified as thermophilic microorganisms, but these results suggest that this physiological criterion is perhaps not decisive in the characterization of both bacterial families. The presence of members of these 6 families in anoxic or micro-oxic conditions is consistent with the lifestyle of these microorganisms at the interface with the steel of the pipeline, the corrosion of which can release H_2_ as energy and electron sources, for example (Rajala et al., 2017). In the water samples taken with the downhole samplers, 54 OTUs were found, including 11 not highlighted from the WHS samples. Seven OTUs had a higher abundance of between 89 and 95.6% of all the OTUs detected in these samples. These main OTUs are affiliated with *Rhizobiaceae* (Cluster_1), *Burkholderiaceae* (Cluster_2), *Desulfurivibrionaceae* (Cluster_5), *Sphingomonadaceae* (Cluster_6), *Pseudomonadaceae* (Cluster_7 and Cluster_13), *Moraxellaceae* (Cluster_8), and an unknown family (Cluster_9). These families have already been found in other deep continental anoxic environments and have been supposed to be involved in sulfur and nitrogen cycles (Mu et al., 2014; Chiriac et al., 2018; Nuppunen-Puputti et al., 2018; Soares et al., 2019; Bell et al., 2020). Because some of these families include bacteria described to be aerobic or nitrate-reducing, being certain of the quality of the sampling is essential. Since deep aquifers are free of O_2_ and NO_3_^-^, these families are often considered as surface and/or soil contaminants during the sampling from deep aquifers (Pedersen et al., 1997; Kadnikov et al., 2020). These microorganisms can adapt and survive in anoxic oligotrophic conditions without terminal electron acceptors such as O_2_ and NO_3_^-^, probably by fermenting the organic molecules trapped in clays and released during the displacement of formation water during sampling. Our understanding of microbial environments at great depths is still limited. Samplings must be irreproachable so as not to add doubt to results that can sometimes be surprising. Thus, cyanobacteria and photosynthetic microorganisms were discovered in a 613-m-deep borehole from aseptic subsamples of cores (Puente-Sánchez et al., 2018). Using metagenomic approaches, the authors explained the ability of these organisms to colonize the deep continental subsurface, a light-deprived environment, by possible lithoautotrophic growth based on hydrogen.

**Table 1:** Alpha-diversity values of microbial community in the formation water collected at the wellhead (WHS) and with the downhole samplers (DS) after a biomass concentration with filters with a porosity of 0.22 μm and 0.1 μm from the 0.22 μm-eluate.

	Observed	Chao	Shannon	InvSimpson
DS1_0.1 μm	32	32.25	2.2026627057447	6.68692475963143
DS1_0.22 μm	16	16	1.6071602891354	4.06101605909724
DS2_0.1 μm	35	36.5	1.69487857291484	4.00962888083069
DS2_0.22 μm	16	16	1.05375703144571	2.1445596371457
WHS1_0.1 μm	54	55.4285714285714	0.919883527901311	1.52772507061242
WHS2_0.22 μm	37	39	0.506904400990633	1.22670219064637
WHS2_0.1 μm	43	46	1.35404072016678	2.12060845612808
WHS2_0.22 μm	34	34.25	0.751805115394659	1.4350021942113
WHS3_0.1 μm	39	41	1.83916566155245	3.587061073388
WHS3_0.22 μm	56	58.3333333333333	1.55171796086355	2.97583926302589
WHS4_0.1 μm	24	25	0.739682176064375	1.3862512153454
WHS4_0.22 μm	25	25	2.05107884523552	4.46583265503757

## Conclusion

Similar to surface ecosystems, deep aquifers are shaped by the living organisms that develop there. These ecosystems are by nature oligotrophic and therefore poor in energy. Their use in the storage of energy or CO_2_ modifies their physicochemical conditions and can be the source of new nutrients and/or energy, increasing the activity of anaerobic heterotrophs, fermenters, and even hydrogenotrophs. The quality of the samples guarantees that the expressed results represent the microbial diversity of the deep subsurface, and the noncontamination of the well from the surface is essential in this regard. Doubt with respect to contamination can result in the classification of certain microorganisms, such as *Pseudomonadaceae*, as contaminants and prevent microbiologists from looking for new possible metabolic pathways that allow them to be maintained in these environments. In this sense, the use of a downhole sampler, common in the study of deep oceanic microbial environments, must be popularized in the study of deep aquifers when wells are used for access. In this paper, the LEUTERT One Phase Sampler OPS sampler commonly used for geoscience studies was successfully prepared to meet the specifics of a microbiological study to collect representative formation waters and was perfectly suited for deployment in areas presenting an explosive risk, such as natural gas storage areas. As we have shown here, the use of this type of sampler allows the elimination of microorganisms present in the well but absent from the original formation water. Finally, the use of a pressurized sampler makes it possible to control depressurization in an optimal manner for the survival of the microorganisms. Depending on the needs of future studies, this protocol can be easily adapted to directly transfer the collected pressurized water from the downhole sampler to a high-pressure reactor or storage in a high-pressure cell.

## Data availability statement

The datasets presented in this study can be found in online repositories. The names of the repository/repositories and accession number(s) can be found in the article/Supplementary material.

## Author contributions

MR-P, SR, FB, ML, HC, PCh, PCé, and AR-P: co-conceived the study. MR-P, MG, PH, and AR-P: carried out sampling and microbiological studies. MR-P, MG, FB, ML, HC, and PCh participated in the preparation of the downhole sampler and the sampling. All authors contributed to interpretation of results and paper writing.

## Funding

Storengy and Teréga are acknowledged for funding this research project. MR-P salary was supported by E2S-UPPA.

## Conflict of interest

DD is employed by STORENGY – Geosciences Department. PCh and GC are employed by Teréga. SR, FB, and ML are employed by Modis.

The remaining authors declare that the research was conducted in the absence of any commercial or financial relationships that could be construed as a potential conflict of interest.

## Publisher’s note

All claims expressed in this article are solely those of the authors and do not necessarily represent those of their affiliated organizations, or those of the publisher, the editors and the reviewers. Any product that may be evaluated in this article, or claim that may be made by its manufacturer, is not guaranteed or endorsed by the publisher.

## Footnotes

^1^ https://deepcarbon.net/

## Supplementary material

Supplementary TABLE S1 Taxa of the OTUs presented in Figure 3 (heatmap).

Supplementary Figure S1 Sterilization of the sampler using a heating cell. On the left, a photograph of the heating cell. On the right, thermal imaging during the sterilization step at 125 °C for two hours in the presence of distilled water.

Supplementary Figure S2 Pressure compensation system for precisely controlling the depressurization of the downhole sampler. On the left, a schematic representation of the sampler; on the right, a sterilized high-pressure manual pump equipped with a pressure gauge and one two-way valve.
