# Supplementary figures and images for: A deep continental aquifer downhole sampler for microbiological studies
Source: Front Microbiol. 2023 Jan 4;13:1012400. doi: 10.3389/fmicb.2022.1012400 (PMC9846368; doi:10.3389/fmicb.2022.1012400)

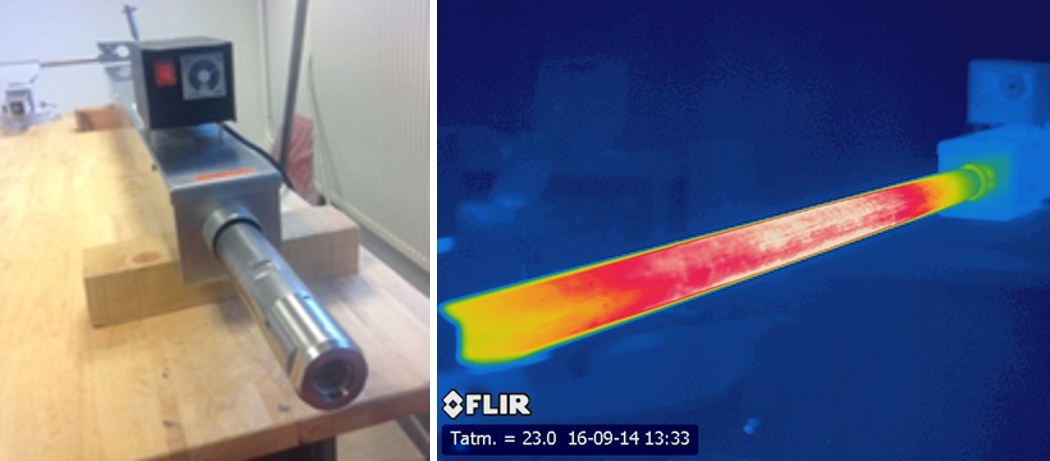

Supplement: Supplementary Figure S1 — Sterilization of the sampler using a heating cell. On the left, a photograph of the heating cell. On the right, thermal imaging during the sterilization step at 125 °C for two hours in the presence of distilled water. [file Image_1.TIF]

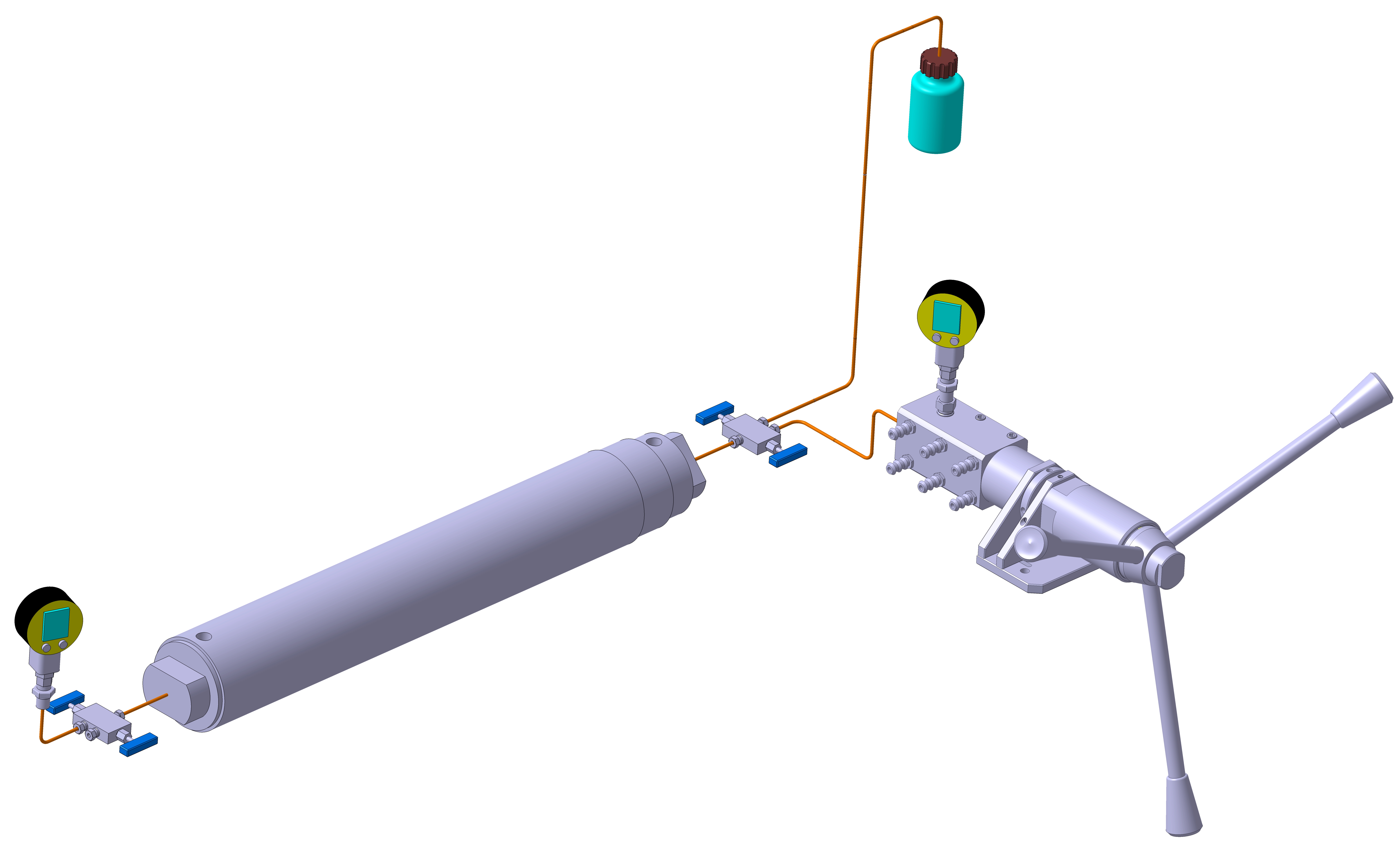

Supplement: Supplementary Figure S2 — Pressure compensation system for precisely controlling the depressurization of the downhole sampler. On the left, a schematic representation of the sampler; on the right, a sterilized high-pressure manual pump equipped with a pressure gauge and one two-way valve. [file Image_2.JPEG]
